# Prevention of vasculitis and vascular pain by side route administration of vinorelbine: A case report

**DOI:** 10.1002/ccr3.7258

**Published:** 2023-05-04

**Authors:** Shunichi Ishii, Takenori Ichimura, Daisuke Ichikura

**Affiliations:** ^1^ Department of Hospital Pharmaceutics School of Pharmacy, Showa University Tokyo Japan; ^2^ Department of Pharmacy Services Showa University Northern Yokohama Hospital Kanagawa Japan

**Keywords:** drug administration route, lung neoplasms, vasculitis, vinorelbine

## Abstract

A 49‐year‐old female with non‐small‐cell lung cancer was placed on adjuvant chemotherapy with vinorelbine (25 mg/m^2^: Day 1.8) and cisplatin (80 mg/m^2^: Day 1). The simultaneous intravenous infusion of vinorelbine from the side route and 500 mL of saline from the main route successfully prevented vasculitis and vascular pain.

## Abstract 2

The simultaneous intravenous infusion of VNR from the side route and 500 mL of saline from the main route prevented VNR‐induced vasculitis and vascular pain.

## 1 INTRODUCTION

Vinorelbine (VNR) is a necrotic anticancer drug that causes severe pain even with a small amount of extravasation that may progress from skin symptoms such as redness, swelling, blisters, and necrosis to ulcer symptoms. VNR has a low pH (3.3–3.8), suggesting that vasculitis and vascular pain may occur due to the exposure of the blood vessel wall and irritation of the intima during its administration.^1^ In addition to pain caused by external leakage, symptoms such as vasculitis and vascular pain are common. The frequency of vasculitis and vascular pain due to VNR is high. Moreover, major adverse events of VNR include Grade 3 vasculitis, which occurs in 5% of patients.^2^ Due to the lack of a standardized preventive method, various preventive modalities are adopted in different institutions. There is currently insufficient evidence for effective preventive measures to alleviate acidity, which is considered to be the cause of vasculitis and vascular pain.

We experienced a case of vasculitis and vascular pain after VNR administration and, as it was considered to be the effect of the contact of high concentrations of VNR with the blood vessel, we infused 500 mL of physiological saline intravenously over 2 h as a preventive measure through the main route and VNR from the side route for 6 min. We report a case in which we were able to prevent the symptoms of vasculitis and vascular pain during intravenous VNR infusion.

## 2 CASE HISTORY

A 49‐year‐old female patient underwent medical checkup at a clinic in mid‐July 2021. Her chest radiograph showed an abnormal shadow in the right lower lung field. On computed tomography (CT) examination, a tumor was seen in the corresponding area and the patient was referred to the internal medicine department of Showa University Northern Yokohama Hospital. Chest CT and positron emission tomography showed a nodule, approximately 26 mm in diameter and an enlarged interlobular lymph node in the lower right anterior lung, suggesting primary lung cancer. Upon admission, the investigation results revealed no impaired glucose tolerance, tumor markers, or any other abnormal blood or biochemical test reports.

### 2.1 Differential diagnosis, investigations, and treatment

Based on imaging and bronchoscopy findings, the patient was diagnosed with non‐small‐cell lung cancer, clinical stage IIa (T1N1M0). On August 2021, thoracoscopic right lower lobe resection and lymph node dissection were performed. In September 2021, the first course of cisplatin (CDDP) + VNR therapy (CDDP 80 mg/m^2^: Day 1 + VNR 25 mg/m^2^: Day 1.8: every 3 weeks) was started as postoperative adjuvant chemotherapy.^3^, ^4^ Fluids were adequately administered during the VNR administration on Day 1 of the first course. During the first 7 days of the first course, no symptoms of vasculitis or vascular pain were observed. However, after the eighth day of the first administration, vasculitis and vascular pain were manifested to an extent that was not tolerated by the patient. Moreover, Grade 2 adverse events (Grade 2 General disorders and administration site conditions—Other, specify, Common Terminology Criteria for Adverse Events) appeared, and the symptoms persisted for 1 week, extending over the forearm and upper arm on the side of administration. The second course was the same as the first course. In this case, no extravasation was confirmed by intravenous drip infusion including VNR in each course.

**FIGURE 1:**
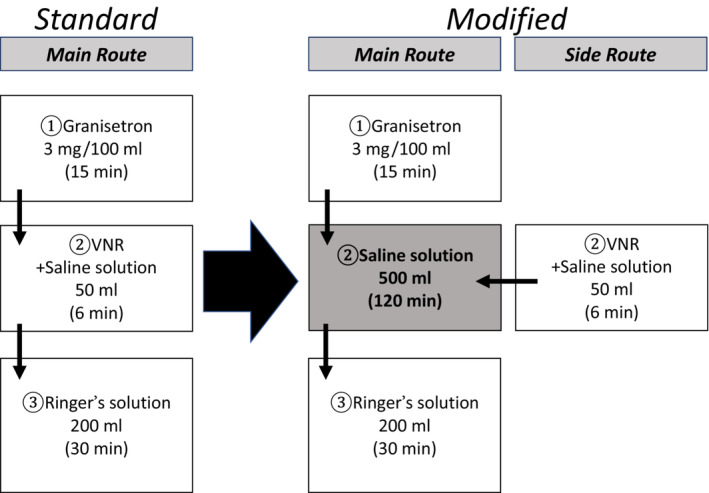
Method of dosage on Day 8.

To prevent vasculitis and vascular pain symptoms, the third course of CDDP + VNR regimen was administered on Day 8 by intravenous drip infusion of 500 mL of saline through the main route over 2 h as a preventive procedure and VNR through the side route over 6 min (Figure 1). The modified regimen was described as a preventive method.

### 2.2 Outcome and follow‐up

As a result of this simultaneous administration of saline, no symptoms of vasculitis and vascular pain appeared after Day 9 of the third course, or even in the fourth course. All four courses of postoperative adjuvant chemotherapy could be completed. Patient examination revealed no vasculitis or vascular pain.

## 3 DISCUSSION

With the aim of preventing the onset of vasculitis and vascular pain due to VNR, and reducing the irritation of the vascular endothelium, 500 mL of physiological saline (our modified regimen) was intravenously infused over 2 h through the main route as a preventive method, and VNR was administered through the side route over 6 min. These findings suggest that the preventive method of concurrent intravenous drip infusion may prevent the onset of vasculitis and vascular pain.

It has been reported that vasculitis caused by VNR can be alleviated by shortening the infusion time of VNR to within 6 min^5^; however, vasculitis still occurs in approximately 10% of patients. In our modified regimen, the administration rate of VNR was originally for 6 min, and it is considered that the effect of the administration rate on vasculitis is small. In addition, local irritation due to VNR was confirmed by a study performed in vivo. The administration of VNR into the auricular vein in rabbits led to a high local irritation, shedding light on VNR as a causal factor for vasculitis and vascular pain.^6^

The presented case suggests the effect of intravascular concentrated VNR as a risk factor for vasculitis and vascular pain. The effect of VNR concentration was reported in an vitro study, whereby the transient exposure of vascular endothelial cells to VNR resulted in a concentration‐dependent decrease in VNR in survival of vascular endothelial cells and an increase in the apoptosis‐executing factor caspase 3/7.^7^ Along those lines, decreasing the concentration of VNR through the simultaneous administration of VNR from the side route over 6 min and 500 mL of saline solution from the main route may be a useful method for reducing the concentration of VNR in the blood vessels and preventing vasculitis and vascular pain. In the current case, on Day 1 of the first course, a total of approximately 2000 mL of fluid replacement was provided from the main route to prevent CDDP renal damage; vasculitis and vascular pain did not appear as a result of simultaneous administration of VNR from the side route over 6 min.

However, VNR and saline were also administered sequentially on Day 8 of the first course, but the symptoms of vasculitis and vascular pain appeared from Day 9 of the first course. It is highly possible that the intravascular contact concentration of VNR was the probable cause of vasculitis and vascular pain in this case. With the exception of the factors that caused vasculitis and vascular pain, the intravascular contact concentration of VNR is difficult to examine through this report alone. To establish a method for the safety of VNR administration, including warm compresses and pH regulation, more such cases need to be studied in the future to verify the effectiveness of intravascular VNR dilution by infusion to prevent vasculitis and vascular pain.

## 4 CONCLUSION

It is important to establish a preventive method for vasculitis and vascular pain occurring after VNR administration. Moreover, it is crucial to identify the different risk factors associated with the safe administration of the drug. For a patient who experienced vasculitis and vascular pain along the superficial blood vessels due to VNR administration, 500 mL of physiological saline was administered from the main route, and VNR was administered from the side route over 6 min. With this simultaneous infusion, vasculitis and vascular pain were prevented.

## AUTHOR CONTRIBUTIONS

**Shunichi Ishii:** Conceptualization; data curation; investigation; validation; visualization; writing – original draft; writing – review and editing. **Takenori Ichimura:** Data curation; investigation; validation; writing – review and editing. **Daisuke Ichikura:** Data curation; investigation; validation; writing – review and editing.

## FUNDING INFORMATION

This research received no specific grant from any funding agency in the public, commercial, or not‐for‐profit sectors.

## CONFLICT OF INTEREST STATEMENT

The authors declare that there is no conflict of interest.

## ETHICS APPROVAL STATEMENT

Not applicable.

## CONSENT TO PARTICIPATE

We obtained written informed consent from the patient.

## CONSENT FOR PUBLICATION

Written informed consent was obtained from the patient for anonymized information to be published in this article.

## Data Availability

All relevant data are within the manuscript.
